# Sibling relationships and visual impairment: investigating their bond with a focus on the role of social play

**DOI:** 10.3389/fpsyg.2025.1555895

**Published:** 2025-07-14

**Authors:** Tiziana Battistin, Alessia Zanatta, Vincenzo Zanardo, Luca Brugnaro, Elena Mercuriali, Maria Eleonora Reffo

**Affiliations:** ^1^Department of Neuroscience and Rehabilitation, University of Ferrara, Ferrara, Italy; ^2^Robert Hollman Foundation, Padova, Italy; ^3^Education and Development of Health Care Professions, Azienda Zero, Padova, Veneto Region, Italy

**Keywords:** social play, siblings, visual impairment, sibling relationship, children, relationship quality, blindness

## Abstract

Social play has a key role in the development of social skills in child development, especially in early and middle childhood through peer interaction. Siblings serve very often as children’s first peers, shaping each other’s overall development throughout life. This mixed study investigates the quality of sibling relationships by giving voice to siblings with and without visual impairment, with a focus on the role of social play in their daily lives. Our findings confirm a harmonious relationship quality and the importance of social play in the sibling bond, suggesting that social play may serve as a protective factor for their mental health.

## 1 Introduction

Social play has a crucial role during child development (Ginsburg, 2007; Quesada Zeljkovic et al., 2024), it being relevant for fostering social communication among peers (Leach et al., 2022; Zhao and Gibson, 2022) and representing a key component of children’s social interactions in the early and middle childhood (Quesada Zeljkovic et al., 2024).

Indeed, during social play children develop not only social and communication skills but also concepts and understanding of their socio-cultural context, as proposed quite a long time ago by Vygotskij (1976) and confirmed also more recently (Nicolopoulou, 1993; Leach et al., 2022).

Siblings’ relationships are very often the first peer relationship in a child’s life and typically the longest-lasting, playing a key role in fostering each other’s social development, (Brody, 1998; Howe et al., 2022; Cox, 2023; Shenoy et al., 2024). Siblings, indeed, mutually shape their life histories, their personalities, their learning, and their attitudes from early life, spending a lot of time together and sharing family and social environment (Buist et al., 2013; Edels et al., 2024).

These first relationships are also crucial in determining future mental health and quality of life, serving as a cornerstone for emotional well-being and social development (Shojaee and Alizadeh, 2019; Rawat and Malik, 2024). As highlighted in the meta-analysis by Buist et al. (2013), higher levels of sibling warmth and less sibling conflict are associated with fewer internalizing and externalizing problems.

Literature showed the positive effect of siblings’ social play on socio-communication skills in children with neurodevelopmental disorders, such as Autism Spectrum Disorders (ASD), where these abilities are often impaired (Kossyvaki and Papoudi, 2016; Ben-Itzchak et al., 2019) and represent major diagnostic criteria for this disorder (American Psychiatric Association, 2013). Interventions on Typically Developing (TD) children to promote social play with their siblings with ASD are needed, because of less engagement also of TD siblings in social interactions (Ross and Cuskelly, 2006; Walton and Ingersoll, 2015). Studies have shown that such interventions can be effective in improving siblings’ social interactions in both siblings with and without ASD (Oppenheim-Leaf et al., 2012; Pan et al., 2023). A recent case report (Thomas et al., 2019) showed also the reverse, hence the importance for a child with ASD to teach to his TD sibling in order to increase their social interactions in play.

Children with Attention- Deficit Hyperactivity Disorder (ADHD) also show significant impairments in social interactions, both in family-relationships, including siblings’ ones and in peer interactions (Nixon, 2001; Smith et al., 2002; Nachane et al., 2022). Nixon (2001), in her review, reported how ADHD children are less engaged in social play with their peers, with lower level of reciprocity than non-ADHD dyads. Daffner et al. (2019) demonstrated the importance of sibling-mediated intervention, through social play, to enhance social skills in young children with ADHD.

To our knowledge there is no literature focused on social play in siblings relationships involving children with and without Visual Impairment (VI), even if there are two articles, our previous one (Battistin et al., 2024) and the contribution by Erdem et al. (2024), which describe social play as one of the features of sibling relationships in the context of VI, without considering it a central topic of investigation. Visual Impairment is a permanent reduction in visual function and can be caused by ocular diseases and/or genetic factors affecting the eye or by Cerebral Visual Impairment (Fonteyn-Vinke et al., 2022). VI impacts on the development of social skills (Gui et al., 2023; Carnevali et al., 2022) and of motor development, which has a key role in driving the development of cognitive functions (Veldman et al., 2019) and in being fundamental for sensory-motor play which is an integral part of early relationships (Adolph and Hoch, 2019). Children with VI rely primarily on their other senses (Houwen et al., 2022) and usually follow atypical developmental trajectories. From the earliest stages of life, they may face challenges in forming attachment bonds and in communicating with caregivers, often due to the lack of eye contact, which hinders the natural course of early interactions (Battistin et al., 2024; Gui et al., 2023). Research highlights that the quality of early caregiver-infant relationships, supported by visual feedback, is a key predictor of future cognitive and socio-emotional development (Grumi et al., 2021). Moreover, during early caregiver-child interactions, children with VI show reduced non-verbal communicative and expressive behaviors, such as facial mimic and body language, compared to their sighted peers (Grumi et al., 2021). Vučinić et al. (2013) analyzed protective and risk factors for social interactions in children with VI, highlighting how these children often have fewer friends and social interactions and how this aspect leads to emotional-behavioral problems.

In our previous qualitative study (Battistin et al., 2024), findings revealed that the main characteristics of being siblings of children with VI are their unconditional love and readiness to help, together with feelings such as empathy, friendliness, sorrow, and sadness.

With an extension of this study, we aimed at evaluating the quality of their relationship, giving voice to both siblings of the dyad in order to explore and analyze the role of social play in their daily lives.

This choice arises from our considerable experience at the Robert Hollman Foundation (RHF), a non-profit organization, which offers consultation and support to children with VI and their families. At the RHF, professionals guide educational and rehabilitation activities through social play and whenever possible include sighted siblings.

## 2 Method

### 2.1 Study design

This mixed study is an extension of our previous study of siblings of children with VI, as approved by the RHF Institutional Board (N. R12_2020 RHF).

In this extension study we administered the Sibling Relationship Questionnaire-C revised (SRQ-C, Furman and Buhrmester, 1985; Buhrmester and Furman, 1990; Love et al., 2012) to two groups of siblings (with and without VI) in order to examine whether significant differences existed in the quality of their relationships, particularly, focusing on social play, their prosocial behavior, companionship, and conflict items.

We also investigated the role of social play in daily interactions and relationships, by conducting a semi-structured interview with both siblings with and without VI.

### 2.2 Participants

Our study included two groups: the siblings with VI (VIS) and their sighted siblings (SS; Table 1).

**Table 1:** Demographics of the siblings with and without VI.

Age VIS	Disability (* Added disability)	Birth Order	Age SS
10	Blindness	Third	15
13	Moderate *(Cerebral Palsy)	Twin	13
9	Moderate	Second	11
13	Blindness	First	9
11	Severe	Second	15
9	Blindness	Second	12
8	Moderate	Second	10
14	Blindness	Second	18
11	Moderate	Third	15
13	Moderate	Second	15
8	Blindness	Third	12 17
9	Severe	Twin	11
13	Moderate	First	11
10	Blindness	First	7
8	Moderate	Third	12
14	Severe	First	11
11	Moderate *(Down Syndrome)	Fifth	15
14	Moderate	Second	18
13	Moderate	First	8
10	Moderate	Second	19
10	Blindness	Third	12
14	Blindness	First	9 12

Age of siblings with and without VI is in years and the visual impairment is classified according to the ICD11 (WHO, 2021). * Added disability.

Group 1 (VIS) includes 22 visually impaired siblings, aged 8–14 years (12 Males; *M* = 11,14; SD = 2,17) with different degrees of VI, from moderate low vision to blindness (WHO, 2021). According to ICD 11 (WHO, 2021), moderate VI is diagnosed when visual acuity (VA) is equal or better than 1/10 and worse than 3/10; severe VI when VA is equal or better than 1/20 and worse than 1/10; blindness when VA is worse than 1/20.

Group 2 (SS) includes 23 sighted siblings, aged 7–19 years (9 Males; *M* = 12,69; SD = 3,37).

The two groups were homogeneous for age (*t*-student test *t* = −1.751; *p* = 0.088).

The selection of children with VI was based on the following inclusion criteria: being under the care of the Robert Hollman Foundation; having at least one sibling; age ≥ 7 years old.

Children with VI and multiple disabilities (any associated disability beyond visual disability, such as cognitive, motor, hearing, behavioral) were included if they were able to answer to the interviewer’s questions.

Inclusion criteria for sighted children and adolescents were: being a sibling of the selected children with VI and being older than 7 years old.

This study took place at the RHF, between the last months of 2024 and the beginning of 2025, and was carried out in accordance with the Helsinki Declaration (World Medical Association, 2013). Parents gave their informed consent to include their children, with and without VI, in the project. All the participants were informed that participation was voluntary and that they could interrupt the interviews at any time.

### 2.3 Procedure

#### 2.3.1 The sibling relationship questionnaire

We administered to the children of both groups the Sibling Relationship Questionnaire (SRQ-C revised, Child version), which is a validated questionnaire to evaluate siblings’ relationships (Furman and Buhrmester, 1985; Buhrmester and Furman, 1990; Love et al., 2012), on a five-point Likert scale (from 1, hardly at all to 5, extremely much) and on four domains Warmth/Closeness, Relative Status/Power, Conflict and Rivalry. We did not administer the Rivalry domain, because it was not related to our aim. The Warmth/Closeness domain includes affection, prosocial behavior, companionship, similarity, intimacy, and admiration of and by the sibling; the Status/Power domain considers dominance and nurturance of and by each sibling in the dyad; the Conflict domain faces quarrelling, antagonism, and competition.

#### 2.3.2 Semi-structured interview

In this extension study, we conducted a semi-structured interview, lasting 15–25 min, with a child-friendly approach. Siblings in both groups (VIS and SS) recounted how they spent their daily routines and what the characteristics of their social play were, expressing also their thoughts and feelings. The interviews were performed in person at the RHF with children with VI (Group 1 VIS) and online for their siblings (Group 2 SS), due to the difficulties for quite a few of them to come in presence. Children were alone during the interviews, in order to be free to talk, without any possible parental influence. We made this choice based on ‘the new sociology of childhood’, chosen as a conceptual framework already in the previous article (Battistin et al., 2023), because it allows children to be active agents, therefore considered autonomous and competent in expressing their own opinions and feelings (Prout, 2011). Questions were repeated, if required and clarifications on terms were provided if necessary. The entire interview respected the child’s time without any insistence on answering. Three open questions were selected from the interview of the previous study (Battistin et al., 2023), while the other 15 questions were identified as being simple, easy to answer and based on siblings’ everyday situations when delving into social play.

All interviews were audio-recorded and fully transcribed with the help of two colleagues and with prior informed consent of the family. Both researchers added their own notes during the interviews, regarding non-verbal (facial mimic, muscle tone, posture) or paralinguistic behaviors (voice tone, emphasis, pauses, rhythm) in order to use them, in the qualitative content analysis, as a useful tool (Vaismoradi et al., 2016).

In Table 2, questions are reported.

**Table 2:** Questions of the semi-structured interview.

How do you spend your time at home? Do you play alone or with your sibling? What games do you play? Do you lend your toys to your sibling? Do you borrow toys from them? Do you prefer active games, or quiet games (i.e., board games)? Do you prefer playing with your sibling or with friends? If you play with your sibling, is it indoors or outdoors? If outdoors, do you play more often in the garden, at the park, or at a friend’s house? Do you play role-playing games together? Which ones? How important is and has sibling play been for you? How much time do you spend playing together? When you play together, is there someone who usually leads the game? Who? When you are playing together, do you invent new games? Which ones? Do you tell your friends about the games you play with your sibling? Do you like joking or playing pranks during games? Do you like teasing during games? Do you like talking while playing? Do you express your emotions (e.g., joy, anger…) while playing? Do you play with your friends and include your sibling too? Do you help your sibling while playing?

### 2.4 Data analysis

#### 2.4.1 Statistical analysis

Descriptive statistics, reported as mean, median, and standard deviation, were utilized. *U*-Mann–Whitney test was performed to investigate if there were any statistically significant difference in the two groups of siblings; in particular we were mostly interested in cooperating, sharing, and participating in play activities, as well as in conflict behavior. Linear regression analysis was applied to evaluate the impact of visual impairment and of age (two subgroups were created according to age ≥ or < 13 years) on the SRQ scores. All analyses were carried out using the statistical software Jamovi ver. 2.6 (Jamovi Project, 2024) and the R statistical software, vers. 4.4 (R Core Team, 2024).

Cronbach’s alpha (*α* = 0.893) was performed to measure their reliability, that is, to verify the reproducibility of the results provided.

#### 2.4.2 Qualitative analyses

We used content analysis to examine the interview responses. Content analysis is a qualitative analysis approach to examine narrative material (Duriau et al., 2007; Vaismoradi et al., 2013). This approach allows both the qualitative interpretation of data and the quantification of codes and offers an analytical flexibility too. This process starts with an initial phase/step of familiarization with the data, during which we engage deeply with the interview transcripts in order to derive codes and subsequently to create themes. After transcribing the audio-recordings, we integrated them with our observational notes regarding non-verbal and paralinguistic behavior, highlighting them in brackets, to obtain a final version of each interview for analysis. These final transcripts were again compared to the recordings to verify accuracy and correct insertion of notes. They were also actively read several times by both researchers to familiarize themselves with the data. Notes were begun on these final drafts to make sense of the data for the subsequent coding process.

We conducted the systematic coding process independently, using highlighters to underline and identify units of meaning. We read the transcripts multiple times, paying attention to each piece of data and its relevance to the research question. At the end of this coding process, we discussed and compared our codes and generated the main themes together.

## 3 Results

Descriptive analysis of each question is shown in Table 3.

**Table 3:** Descriptive statistics of SRQ-C single items.

Question	Group	*N*	Mean	Median	SD	*p*
Q1	SS	23	3.48	3.00	0.846	0.100
	VIS	22	3.00	3.00	0.816	
Q2	SS	23	3.83	4.00	0.887	<0.001*
	VIS	22	2.41	2.00	1.260	
Q3	SS	23	3.26	3.00	1.251	0.944
	VIS	22	3.32	3.00	1.129	
Q4	SS	23	3.00	3.00	1.243	0.576
	VIS	22	2.82	3.00	1.006	
Q5	SS	23	2.96	3.00	1.364	0.494
	VIS	22	2.68	3.00	0.894	
Q6	SS	23	3.83	4.00	0.834	0.019*
	VIS	22	3.14	3.00	1.037	
Q7	SS	23	3.30	3.00	0.974	0.552
	VIS	22	3.45	4.00	1.184	
Q8	SS	23	2.39	2.00	1.305	0.255
	VIS	22	2.82	3.00	1.220	
Q9	SS	23	3.00	3.00	0.953	0.051
	VIS	22	2.45	2.00	0.800	
Q10	SS	23	3.22	3.00	1.166	0.032*
	VIS	22	2.45	2.00	1.101	
Q11	SS	23	2.48	2.00	1.310	0.480
	VIS	22	2.27	2.00	1.386	
Q12	SS	23	3.87	4.00	0.920	0.143
	VIS	22	3.41	3.00	1.054	
Q13	SS	23	3.65	4.00	0.982	0.103
	VIS	22	3.14	3.00	1.082	
Q14	SS	23	2.61	3.00	1.076	0.199
	VIS	22	3.09	3.00	1.269	
Q15	SS	23	3.22	3.00	0.998	0.090
	VIS	22	2.73	3.00	0.935	
Q16	SS	23	3.83	4.00	1.029	0.006*
	VIS	22	2.77	3.00	1.270	
Q17	SS	23	2.83	3.00	1.337	0.478
	VIS	22	3.09	3.00	1.192	
Q18	SS	23	2.74	3.00	1.096	0.596
	VIS	22	2.91	3.00	1.065	
Q19	SS	23	2.87	3.00	1.456	0.898
	VIS	22	2.77	3.00	1.066	
Q20	SS	23	4.52	5.00	0.730	0.007*
	VIS	22	3.86	4.00	0.834	
Q21	SS	23	3.91	4.00	1.164	0.071
	VIS	22	3.27	3.00	1.202	
Q22	SS	23	4.48	5.00	0.730	<0.001*
	VIS	22	3.68	4.00	0.716	
Q23	SS	23	2.91	3.00	0.848	0.748
	VIS	22	2.86	3.00	0.834	
Q24	SS	23	2.65	2.00	1.301	0.298
	VIS	22	2.27	2.00	1.316	
Q25	SS	23	2.35	2.00	1.335	0.207
	VIS	22	2.82	3.00	1.332	
Q26	SS	23	4.26	5.00	0.864	0.032*
	VIS	22	3.55	4.00	1.143	
Q27	SS	23	3.91	4.00	0.848	0.141
	VIS	22	3.45	3.50	1.057	
Q28	SS	23	3.00	3.00	1.206	0.797
	VIS	22	3.09	3.00	1.231	
Q29	SS	23	3.09	3.00	0.949	0.895
	VIS	22	3.00	3.00	1.069	
Q30	SS	23	3.65	4.00	0.982	0.015*
	VIS	22	2.64	2.00	1.399	
Q31	SS	23	3.17	3.00	1.154	0.421
	VIS	22	2.91	3.00	1.342	
Q32	SS	23	2.26	2.00	1.096	0.472
	VIS	22	2.55	2.00	1.262	
Q33	SS	23	2.57	2.00	1.273	0.468
	VIS	22	2.82	3.00	1.220	
Q34	SS	23	3.91	4.00	0.848	0.073
	VIS	22	3.45	3.00	0.800	
Q35	SS	23	3.00	3.00	1.044	0.953
	VIS	22	3.00	3.00	0.976	
Q36	SS	23	2.70	3.00	1.222	1.000
	VIS	22	2.68	3.00	1.249	
Q37	SS	23	2.87	3.00	1.100	0.886
	VIS	22	2.91	3.00	1.109	
Q38	SS	23	2.43	2.00	1.037	1.000
	VIS	22	2.41	2.50	1.182	
Q39	SS	23	2.35	2.00	1.152	0.394
	VIS	22	2.68	2.00	1.249	
Q40	SS	23	4.17	4.00	0.778	0.015*
	VIS	22	3.55	3.00	0.858	
Q41	SS	23	3.91	4.00	0.793	0.075
	VIS	22	3.45	3.00	0.858	
Q42	SS	23	3.35	3.00	0.935	0.161
	VIS	22	2.95	3.00	0.950	

Q = question; N = number of siblings in each group; *p-value <0.05.

No significant differences were found between the two groups in the prosocial behavior, evaluated in the questions: 1 (doing nice things for each other), 15 (cooperation), 29 (sharing), as well as in the companionship questions: 7 (doing things together), 21 (playing and having fun with each other), 35 (spending time together).

A statistically significant difference was found between the two groups, with SS making higher scores, in the responses to the questions 2 (*p* < 0.001; showing their sibling how to do things they do not know how to do), 6 (*p* = 0.019; caring about each other), 10 (*p* = 0.032; telling each other everything), 16 (*p* = 0.006; helping with things they cannot do by themselves), 20 (*p* = 0.007; loving each other), 22 (p < 0.001; meaning to each other), 26 (p = 0.032; looking up to and feeling proud of their sibling), 30 (*p* = 0.015; teaching their sibling things they do not know), 40 (*p* = 0.015; thinking highly of their sibling).

Furthermore, the mean score for Warmth/Closeness, which reflects the average score of the seven subscales, was higher in SS (*M* = 3.53; DS = 0.59) compared to VIS (*M* = 3.11; SD = 0.45). The mean score for Conflict, which reflects the average score of the three subscales, was lower in SS (*M* = 2.65; SD = 0.36) compared to VIS (*M* = 2.80; SD = 0.27).

The mean score for Relative Status/Power, which is calculated by the sum of the subscales ‘nurturance of sibling’ and ‘dominance of sibling’ minus the sum of the subscales ‘nurturance by sibling’ and ‘dominance by sibling’, resulted positive in SS (+1.65) and negative in VIS (−1.50).

Results from linear regression analyses are shown in Table 4.

**Table 4:** Linear regression analysis.

SRQ-C domain	Predictor	Estimate	SE	*t*	*p*
Warmth and closeness	Intercept	90.179	8.283	10.89	<0.001
	AGE	−0.997	0.628	−1.59	0.120
	VIS – SS	–10.577	3.613	−2.93	0.005*
Status/Power	Intercept	47.695	5.519	8.64	<0.001
	AGE	−0.852	0.418	−2.04	0.048*
	VIS – SS	–4.529	2.407	−1.88	0.067
Conflict	Intercept	24.669	4.926	5.008	<0.001
	AGE	−0.274	0.373	−0.733	0.467
	VIS – SS	0.789	2.148	0.367	0.715
Sum-score	Intercept	162.54	13.22	12.30	<0.001
	AGE	−2.12	1.00	−2.12	0.040*
	VIS – SS	–14.32	5.77	−2.48	0.017*

*p-value <0.05.

The domain Warmth/Closeness is significantly correlated (*p* = 0.005) to the group category, showing that the SS expressed in the interview more warmth and closeness than VIS.

The domain Relative Status/Power is significantly correlated (*p* = 0.048) to age, indicating that nurturance and dominance is higher in SS and decreases with increasing of age in the subgroup ≥13 years old. It is not correlated to the group category, even if the *p* = 0.067 indicates a marginal trend toward statistical significance.

No differences were shown in the Conflict scales between the two groups (*p* = 0.715) and in relation to age (*p* = 0.467).

The sum-score of the three domains is significantly correlated both to group category (*p* = 0.017) and to age (*p* = 0.040).

Analyzing the semi-structured interviews, the first emerging theme was “Social play and growth.” Siblings referred to playing with their sibling in the present and in the past: the young children told about their daily play with their siblings and the oldest ones remembered the nice memories of their childhood when they spent more time playing together, such as in the quote: *“I play daily with my brother; when we were younger we loved playing with Lego, first building towns and then destroying them (smile and laugh), now we moved mostly from games to video games, always together!”* (VIS, 14 years old).

These recollections highlighted the crucial role that social play had – and continues to have – in their daily lives. A great majority of them, in both groups (VIS 82% and SS 87%), confirmed this answering to question 8. Some siblings made it clear how social play had a key role in their development, as in the following quotes: *“Playing helped us in our development”* (VIS, 14 years old); “*Playing with my sisters was very important because it helped us grow together and feel more connected”* (SS, 15 years old); sighted siblings also highlighted how their sibling’s visual impairment influenced their development, as in the following quote: *“My sister has always been a source of pride rather than someone to hide. I also feel like I’ve matured earlier; having a blind sibling opens your eyes to different perspectives” (SS, 18 years old).*

The second theme was “Siblings versus friends,” which highlighted the depth of the sibling bond and the boundaries that distinguish it from friendship – in terms of what is allowed to share and what is exclusive to the sibling relationship. Both their verbal answers and non- verbal behavior showed the significant role that their experiences of social play had on their bond, as seen in these quotes: *“If it wasn’t for my sibling I would not even have played”* (VIS, 13 years old); *“Playing with my sister is very important because it makes me feel like an important person in her life. Sometimes I notice that her classmates pretend to be her friends, I do not pretend.”* (SS, 15 years old) or *“My brother makes me happy! When he gets hurt, I cuddle him”* (VIS, 8 years old) or *“It’s always nice when we are together!.”*

The majority of siblings spend time with their brother/sister, regardless of age, age difference, or daily commitments. It is quite interesting how, in the VIS group, five out of six blind children who initially said they preferred to play alone (question 2), later contradicted themselves in the remaining responses, revealing a significant amount of time and enjoyment shared in sibling play.

Siblings also reported that they enjoy playing with both siblings and friends (especially sighted siblings), but this must occur in different contexts: in fact, only half of VIS and 61% of SS include friends in sibling play. Even fewer, only four children out of 22 VIS (18%) and 30% SS said that they tell their friends about their sibling play. It seems, also from the emphatic tone of their voices, that their bond is experienced as being highly exclusive and that it has to be preserved from any external interference. The great majority of siblings were very categorical in answering “No” to question 12; the reasons of such a response were mostly related to the private and familiar nature of sibling play such as in these quotes: “*No, we prefer to keep it between us. The same, I do not tell my brother the games I play with my friends*.” (VIS, 9 years old) or “*No! This is my business!*” (VIS, 14 years old). A few siblings made it clear that they think their friends do not care, such as in the following quote: *“I do not think anyone cares if I tell them what I do with my sister*” (SS, 11 years old) or *“I do not think anyone gives a damn”* (VIS, 13 years old) or *“No, my friends do not care about it.”* (VIS, 9 years old).

A third theme is “Featuring social play.” When we analyzed the types of games played, we found a significant difference between the two groups: almost all the sighted siblings (91%) strongly preferred active games, whereas in the group of children with VI, the responses were divided, showing a preference for active games among children with low vision and a preference for board games and other quiet games among blind children. These differences are understandable given the nature of the visual disability, which inevitably influences access, comfort, and confidence in certain types of play. A few children also were able to express verbally their dislike for some games because of their difficulty due to visual disability, such as in the following quote: *“I do not like tag because I’m not fast at running and I get teased for my slowness.”* (VIS, 9 years old).

Social pretend play was reported by nearly all siblings in both groups (82%); the detailed description of all the roles they played and their enthusiasm in telling us reflects its relevance in their relationship, as in the following quote: *“We play that I’m someone who write songs in braille, we record them on my brother’s phone and we practice because when I grow up I want to be a rap singer!!!”* (VIS, 9 years old).

Exploring their favorite play environment, the majority of siblings prefer to play mostly outdoors, even if 70% of VIS preferred their garden at home, which is always a protected environment; the oldest siblings, in both groups, preferred indoors, for playing videogames or listening to music together. Seven children nevertheless, all blind, showed a preference for indoors. Sighted siblings demonstrated varying levels of awareness of their sibling’s visual impairment, depending on their age. Many of them showed an adaptive and supportive approach to play, modifying their activities to accommodate their sibling’s needs. For instance, one SS, 10 years old said: “*I have fun with my sister, but since she cannot play much outdoors, we invite some friends at home to play together.”* or *“We cannot play with the ball because she’s scared* but *I’m very happy to change game if this helps her”* (SS 11 years old).

The majority of siblings (59% VIS and 87% SS) also said that they invent new games or modify what they are playing. 70% of both groups respect the rules of the game; in both groups the majority of siblings like joking during the play, even if there is quite a difference between the two groups (87% SS and 59% VIS). What is certain is that a great percentage of them, in both groups (73% VIS and 70% SS), do not like teasing at all during the game.

Verbal dimension was indicated as important because 91% of siblings in both groups like talking during play.

The last theme is “Emotions and Feelings”: in both groups the majority of siblings (83% SS and 59% VIS) express their emotions when playing together, even if in the VIS group nine siblings said clearly they do not express themselves, as in the following quotes: *“No, not at all, I hold back what I feel”* (VIS, 7 years old) or *“I do not express my feelings, I always keep them with myself, it’s my choice”* (VIS, 11 years old) or *“No, I never do it!”* (VIS,14 years old).

Other feelings were unmasked by siblings of both groups, highlighting how VI impacts on their relationship, such as in the quotes: *“My sister is ashamed of me when she plays with her friends”* (VIS, 9 years old) or *“My sister (unlike me) never had any difficulty in playing!”* (VIS, 11 years old) or *“My sister (unlike me) is always successful in everything she does.”* (VIS, 8 years old) or *“I always used a lot of irony about his disability so that he could cope better with it.”* (SS, 18 years old) or *“When he wins, I feel well seeing him happy”* (SS; 13 years old).

## 4 Discussion

Results from the administration of the SRQ-C indicate a harmonious sibling relationship quality with higher levels in Warmth and lower in Conflict in both groups; however, SS showed a significantly higher level of positivity (Warmth/Closeness) and a lower level of negativity (Conflict) than VIS. In the Prosocial, Companionship and Conflict items, related to social play, no significant differences were found between the two groups. The domain Relative Status/Power resulted significantly correlated to age, with a positive mean score observed in SS and a negative one in VIS; this suggests that VIS believe SS have greater dominance and nurturance in their relationship.

Findings from the semi-structured interviews show how social play is relevant to siblings, being an enriching construct in their growth and mutually molding their relationship and how it is experienced as an exclusive bond. Siblings reported their preferences about social play, such as preferring active games outdoors, social pretend play, joking, and talking during playing. Siblings mostly express their emotions and feelings while playing, even if quite a few VIS siblings prefer to keep within themselves.

These results and findings are rich in details, complex to interpret, and even not yet fully explainable because more research on this topic is needed. Indeed, it is the first time that social play has been analyzed in depth in dyads of siblings with and without VI.

The relevance of social play in child development as well as in sibling relationships is extensively described in literature (Ginsburg, 2007; Howe et al., 2022; Leach et al., 2022; Quesada Zeljkovic et al., 2024). Our findings confirmed its importance and highlighted the exclusively private nature of social play in the bond between siblings, especially in VIS, who tend to keep it separate from play with friends. This may be due to their shared life history, characterized by many emotionally intense interactions and experiences rooted in familiarity; in particular for VIS, there is also a strong sense of blind trust in their sighted siblings, who are seen as trustworthy role models and serve as sources of support and safety when needed (Howe et al., 2022; Battistin et al., 2023; Erdem et al., 2024). Literature has also documented challenges in social interactions and fewer peer relationships among children with VI (Vučinić et al., 2013; Manitsa and Doikou, 2022), for whom sighted siblings often represent a key pillar in daily play and social engagement. This is confirmed also by the negative mean score in Relative Status/Power in VIS, which shows how VIS have less control and nurturance in their relationship, relying on their sighted siblings. The significant differences found in the answers to questions 2, 16 and 30 of this domain, also confirm the role of the carers of SS independently from age, which has been already described by Erdem et al. (2024), Hemati Alamdarloo et al. (2019) and in our previous paper (Battistin et al., 2023). In the current study this role is experienced positively by SS, not affecting the quality of their sibling relationship. In fact, results from the SRQ indicate a harmonious sibling relationship quality with high level of Warmth/Closeness and low levels of Conflict in both groups. No statistically significant differences were found in the Conflict domain, unlike what is reported in the literature for other neurodevelopmental disorders such as ADHD (Nachane et al., 2022; Smith et al., 2002), ASD (Longobardi et al., 2019; Kaminsky and Dewey, 2001) or even sensory disabilities (Hemati Alamdarloo et al., 2019). Conversely the sighted siblings expressed significantly more Warmth/Closeness than VIS, even if not in the subscales linked to social play; this data suggests a tendency to a harmonious and quiet relationship, where also play activities are not characterized by antagonism or arguing/quarrelling. Even if scores in the Warmth/Closeness domain were higher in the SS group, this finding should be interpreted with caution. Data from the semi-structured interviews in fact indicate that VIS group expressed less their emotions and feelings than SS. Therefore, the lower scores may be due not to a less affective component but to a less capability or will to express feelings, including those towards their siblings. Indeed, literature reported more emotional problems in children and young people with VI (Manitsa and Doikou, 2022; Chennaz et al., 2022; Ophir-Cohen et al., 2005), due to the sensory deprivation that affects socio-emotional development from early infancy, impeding or reducing the reciprocal early interactions through eye gaze.

Social play in sibling relationships features a preference even for active and outdoors games; however, the impact of VI is evident, as blind children prefer quieter games at home or in a safe and protective environment such as the home garden. It seems also that the choice of environment is connected to age in both groups: as children grow older, there is a noticeable transition from physically active games to more sedentary activities, such as video games and other forms of home-based play, even if still together. This data confirms what has been partially described also in our previous article (Battistin et al., 2023) and by Erdem et al. (2024). Siblings in both groups also described social pretend play as an ordinary experience in their childhood; this is highly positive in consideration to the difficulties of VI children in social abilities (Caron et al., 2023; Gui et al., 2023) and to the evidence from literature of how this type of play promotes the development of social skills (Fein, 1981; Howe et al., 2022). It is also interesting that both groups highlighted their preference for dialogue during the game, showing how the verbal dimension took on a key role compared to metacommunication which we know to be relevant in social pretend play (de Haan et al., 2021). These data, understandable for VIS as metacommunication is more difficult and reduced due to their visual impairment, show how SS have also adapted their verbal and non-verbal behavior to facilitate their relationship with VIS.

The analysis of these findings, from a psychological perspective, suggests a protective role of social play for the sibling relationship and for their mental health. A study demonstrated how peer play in early years is protective for both externalizing and internalizing problems, suggesting a protective role of social play for children’s mental health (Zhao and Gibson, 2022). Reciprocal sibling interactions during play have been proposed to promote trust and mutual understanding (Howe et al., 2001; Howe et al., 2022). More warmth and less conflict in the sibling relationship would be related to less internalizing and externalizing problems (Buist et al., 2013; Edels et al., 2024).

Our findings also have a clinical implication in providing information for healthcare professionals on possible interventions and one suggestion, drawn from our experience at the RHF, is to include sighted siblings in social play activities to foster their relationship and their development.

Our findings have some limitations: firstly, we only interviewed siblings attending the RHF so there is a possibility of bias, having not considered children from other contexts; secondly, our sample was relatively small, so a larger one should be considered in future studies in order to confirm our findings.

In conclusion, the findings of this study showed that the great majority of siblings in both groups enjoy playing together, have a harmonious sibling relationship quality with more warmth and less conflict, and that social play is significant in their growth and in their sibling relationship. Social play may indeed have a protective effect on their relationships, by being relevant in their daily lives and in terms of fostering shared experiences, which contribute to their well-being.

Future directions could be to conduct a further analysis and comparison of each dyad relationship as well to investigate if there is an effect due to the different degrees of VI.

## Data Availability

The original contributions presented in the study are included in the article, further inquiries can be directed to the corresponding author.
